# Analysis of Gene Expression Patterns of Epigenetic Enzymes *Dnmt3a*, *Tet1* and *Ogt* in Murine Chondrogenic Models

**DOI:** 10.3390/cells10102678

**Published:** 2021-10-06

**Authors:** Judit Vágó, Katalin Kiss, Edina Karanyicz, Roland Takács, Csaba Matta, László Ducza, Tibor A. Rauch, Róza Zákány

**Affiliations:** 1 Department of Anatomy, Histology and Embryology, Faculty of Medicine, University of Debrecen, 4032 Debrecen, Hungary; vago.judit@med.unideb.hu (J.V.); edina.karanyicz@gmail.com (E.K.); takacs.roland@anat.med.unideb.hu (R.T.); matta.csaba@med.unideb.hu (C.M.); ducza.laszlo@anat.med.unideb.hu (L.D.); 2 Department of Medical Biology and Central Electron Microscope Laboratory, Medical School, University of Pécs, 7624 Pécs, Hungary; katalin.kiss@aok.pte.hu; 3 Institute of Biochemistry and Medical Chemistry, Medical School, University of Pécs, 7624 Pécs, Hungary; tibor.rauch@aok.pte.hu

**Keywords:** chondrogenesis, chondrocyte, cell differentiation, C3H10T1/2, micromass culture, mouse embryo, DNA methylation, 5-azacytidine

## Abstract

We investigated the gene expression pattern of selected enzymes involved in DNA methylation and the effects of the DNA methylation inhibitor 5-azacytidine during in vitro and in vivo cartilage formation. Based on the data of a PCR array performed on chondrifying BMP2-overexpressing C3H10T1/2 cells, the relative expressions of *Tet1* (tet methylcytosine dioxygenase 1), *Dnmt3a* (DNA methyltransferase 3), and *Ogt* (O-linked *N*-acetylglucosamine transferase) were further examined with RT-qPCR in murine cell line-based and primary chondrifying micromass cultures. We found very strong but gradually decreasing expression of *Tet1* throughout the entire course of in vitro cartilage differentiation along with strong signals in the cartilaginous embryonic skeleton using specific RNA probes for in situ hybridization on frozen sections of 15-day-old mouse embryos. *Dnmt3a* and *Ogt* expressions did not show significant changes with RT-qPCR and gave weak in situ hybridization signals. The DNA methylation inhibitor 5-azacytidine reduced cartilage-specific gene expression and cartilage formation when applied during the early stages of chondrogenesis. In contrast, it had a stimulatory effect when added to differentiated chondrocytes, and quantitative methylation-specific PCR proved that the DNA methylation pattern of key chondrogenic marker genes was altered by the treatment. Our results indicate that the DNA demethylation inducing *Tet1* plays a significant role during chondrogenesis, and inhibition of DNA methylation exerts distinct effects in different phases of in vitro cartilage formation.

## 1. Introduction

Epigenetics refers to those reversible and heritable biological processes that regulate gene expression without alteration of the primary DNA sequence [1]. Epigenetic regulation is responsible for cell-specific gene expression and for the inheritance of these unique expression patterns to daughter cells [2]. The primary target of epigenetic processes is the DNA-histone complex termed chromatin. The accessibility of a specific DNA segment for transcription factors is influenced by different epigenetic marks, such as DNA methylation or histone modifications [3]. DNA methylation causes gene repression or silencing by adding a methyl group from the ubiquitous methyl donor S-adenosyl methionine to the carbon 5 position of cytosine rings of the DNA, which leads to hypermethylation of a given genomic region. The newly formed unit is called 5-methylcytosine (5-mC). This process is catalyzed by DNA methyltransferases (DNMTs), which can be classified into two groups according to their enzymatic activity. *Dnmt3a* and *Dnmt3b* are de novo methyltransferases that have a role in generating new methylation patterns during ontogenesis. *Dnmt1*, however, has the ability to transfer the already existing methylation motifs during cell division, thus it is referred to as a maintenance protein [4,5]. Methylation sites are exceptionally frequent in the promoter regions of genes because they contain numerous CpG sites. Transcription factors are unable to bind to their sites in the case of those that are methylated [6]. Mature cells preserve their DNA methylation characteristics, while differentiating cells can be modulated by demethylating factors during ontogenesis in order to recover the pluripotent characteristics [7]. DNA demethylation is organized chiefly by proteins of the 10-11 translocation methylcytosine dioxygenase (TET) family, which oxidize the methyl group of the 5-mC to 5-hydroxymethyl cytosine (5-hmC), hence reversing the effect of DNMTs and causing hypomethylation [8,9]. Recent findings in murine embryonic stem cells confirmed that *Tet1* and *Tet2* proteins are strongly associated with the O-linked *N*-acetylglucosamine (O-GlcNAc) transferase (*Ogt*) and they act as a complex to maintain the unmethylated CpG-rich DNA regions [10]. *Ogt* is capable of regulating the biological activity of TET enzymes, and has a specific interaction with *Tet1* during developmental processes [11].

Epigenetic regulation is essential during cartilage formation, and DNA methylation is one of the most widely studied epigenetic mechanisms in relation to this developmental process [12,13]. Early stage chondrocyte differentiation is controlled by an array of transcription factors. As an example, SRY-box transcription factor 9 (*Sox9*) is considered as the key transcription factor of chondrogenesis and it is necessary to regulate the expression of cartilage-specific extracellular matrix (ECM) genes [14]. The promoter regions of *Sox9* exhibited a hypomethylated pattern in human synovium-derived mesenchymal stem cells (MSCs) during in vitro chondrogenesis [15]. The cartilage matrix-specific marker gene collagen type II alpha 1 chain (*Col2a1*) was also less methylated in chondrocytes in comparison with fibroblasts [16]. Methylating and demethylating enzymes also play a crucial role in chondrocyte differentiation. Moreover, DNMTs might serve as a promising epigenetic regulatory mechanism in cartilage repair [17]. Previous studies have shown that the chondrogenic differentiation of chicken embryonic limb bud-derived mesenchymal cells is regulated via *Dnmt3a*-specific methylation of the *Sox9* promoter [18]. *Dnmt3b* and *Tet1* were also recognized as significant epigenetic factors in chondrocyte differentiation, transcriptional control of cartilage-related genes, and hypertrophic differentiation of chondrocytes [19,20]. In a recent publication, *Tet1*-mediated *Sox9*-dependent activation of *Col2a1* and *Acan* has been demonstrated during in vitro chondrogenesis of ATDC5 cells [21].

Five-azacytidine (5-azaC) is a compound, which acts as a chemical analogue of the DNA nucleoside cytidine and has the ability to inhibit DNA methyltransferases [22]. Further, 5-azaC significantly promoted the osteogenic differentiation of adult bone marrow-derived murine MSCs [23], which indicates that it may be suitable for targeted control of stem cell differentiation into a desired cell type, for example, chondrocytes. Recent findings show that 5-azaC may also serve as a potential therapeutic agent in the treatment of rheumatoid arthritis [24].

Despite the accumulating wealth of data regarding the epigenetic regulation of gene activity in immature and mature cartilage, there are still many unanswered questions. The impact of epigenetic mechanisms on early stages of chondrogenesis and chondrocyte differentiation has not been described thoroughly, despite their high therapeutic relevance [25,26,27,28]. In this study, we investigated the temporal gene expression patterns of several enzymes influencing DNA methylation during chondrogenesis. We compared data obtained from chondrifying cultures of the murine embryonic mesenchymal cell line C3H10T1/2, murine primary chondrogenic cell cultures, and sections of developing whole mouse embryos. We performed a detailed expression analysis of *Dnmt3a*, *Tet1*, and *Ogt*, and investigated the impact of the inhibition of DNA methylation on chondrogenesis by using 5-azaC. Our results indicate *Tet1* as a prominently expressed gene during both in vitro and in vivo chondrogenesis, and a developmental stage-dependent effect of 5-azaC.

## 2. Materials and Methods

### 2.1. Experimental Models

#### 2.1.1. Primary Chondrifying Micromass Cultures

Micromass cultures were established from mouse limb bud-derived mesenchymal cells following a protocol used on chicken micromass cultures with some modifications [29,30]. First, NMRI laboratory mice were mated overnight. On the following day, successful mating was detected by confirming the presence of the vaginal plug—this day was considered as day 0 of gestation. Embryos on gestational day 11.5 (E11.5) were retrieved from the uterus. NMRI mice were sacrificed according to the ethical standards defined by the University of Debrecen Committee of Animal Research (Permission No. 2/2018/DE MÁB). After some brief washes with sterile calcium and magnesium-free phosphate buffered saline (CMF-PBS), distal parts of fore and hind limb buds were removed and pooled in sterile CMF-PBS. Limb buds were then dissociated in 0.25% trypsin-EDTA (Merck, Kenilworth, NJ, USA) incubated at 37 °C in a CO_2_ incubator (5% CO_2_, 80% humidity) for 20–30 min. After the addition of an equal volume of fetal bovine serum (FBS; Gibco, Gaithersburg, MD, USA), cells were centrifuged for 10 min at 800× *g*. The digested cells were filtered through a 40-μm pore size plastic filter unit (Corning, Tewksbury, MA, USA) in order to gain a single cell suspension of mesenchymal cells. Cells were centrifuged again for 10 min at 800× *g*. The cell pellet was resuspended in high-glucose (4.5 g/L) Dulbecco’s modified Eagle’s medium (DMEM; Sigma-Aldrich, St. Louis, MO, USA) supplemented with 10% (*v*/*v*) FBS, 0.5 mM stabile L-glutamine (Sigma-Aldrich), and antibiotics/antimicotics (penicillin, 50 U/mL; streptomycin, 50 μg/mL; fungizone, 1.25 μg/mL; TEVA, Debrecen, Hungary) at a concentration of 1.0 × 10^7^ cells/mL and 100-μL droplets were inoculated into 35-mm plastic tissue culture dishes (Eppendorf, Hamburg, Germany) to establish micromass cultures. Cells were allowed to attach to the surface for 2 h at 37 °C in a CO_2_ incubator. Finally, 2 mL of culture medium were added into the culture dishes. Cultures were incubated in a conventional cell culture incubator. Day of inoculation was considered as day 0 of culturing. Cell cultures were maintained for 0, 1, 3, 4, 6, 10, or 15 days. The medium was changed on every second day.

#### 2.1.2. Micromass Cultures Established from C3H10T1/2 BMP-2 Cells

The BMP-2-overexpressing C3H10T1/2 cells were maintained as monolayer cultures in high-glucose (4.5 g/L) Dulbecco’s modified Eagle’s medium (DMEM; Sigma-Aldrich) supplemented with 10% (*v*/*v*) FBS, 0.5 mM stabile L-glutamine, 6.6 μg/mL ampicillin, 100 μg/mL streptomycin (TEVA), 5 μg/mL puromycin (Sigma-Aldrich), and kept in a humidified CO_2_ incubator at 37 °C. Once monolayer cultures reached approximately 80% confluence, cells were harvested in order to establish micromass cultures, as described previously [31]. After trypsinisation, cells were centrifuged at 800× *g* for 10 min. The number of cells was determined with the help of an automated cell counter (LUNA^TM^, Logos Biosystems, Annandale, VA, USA). For micromass cultures, cell density was 1.0 × 10^7^ cells/mL. Then, 100-μL droplets of the cell suspension were inoculated into 35-mm plastic Petri dishes (Eppendorf). This day was considered as day 0. After leaving the cells to attach to the surface (2 h, 37 °C), high-glucose DMEM was added to the dishes, which was changed on every second day. The micromass cultures were maintained for up to 0, 5, 10, and 15 days.

### 2.2. PCR Array

Quantitative PCR arrays were used to monitor differential expression of 80 genes encoding epigenetic factors and chondrogenesis markers in micromass cultures established from C3H10T1/2 BMP-2 cells. Total RNA was isolated using a Direct-Zol^®^ RNA Miniprep kit (Zymo Research, Irvine, CA, USA) from cultures harvested on designated days of culturing, and cDNA synthesis was conducted by an iScript RT Supermix kit (Bio-Rad, Hercules, CA, USA) on 1000 ng of total RNA. PrimerQuest software was used to design primers and were manufactured by Integrated DNA Technologies (Coralville, IA, USA). RT-qPCR was performed using a CFX RT-PCR machine (Bio-Rad) and the SsoFastEvaGreen™ Supermix (Bio-Rad). The parameters of PCR were set according to the manufacturer’s protocol. The RT-qPCR primer sequences are listed in Table S1 in the Supplementary Materials. The specificity of the PCR primers was monitored by a post-PCR melting curve analysis, and heatmap analysis was conducted by CFX Maestro software (Bio-Rad).

### 2.3. RNA Isolation and Reverse Transcription

On the designated days of culturing, both primary and cell line-based micromass cultures were washed with physiological NaCl two times and stored at −80 °C. Total RNA from micromass cultures established from C3H10T1/2 BMP-2 cells and primary chondrifying micromass cultures was isolated as previously described [32]. Briefly, cultures were mixed with TRI Reagent (Applied Biosystems, Foster City, CA, USA). Then, 20% RNAse-free chloroform was added and samples were centrifuged at 10,000× *g* at 4 °C for 15 min. After incubation in 500 µL of RNAse-free isopropanol for 1 h at −20 °C, total RNA was dissolved in RNAse-free water and stored at −80 °C. RNA concentration and purity were determined using a NanoDrop 1000 spectrophotometer (Thermo Fisher Scientific, Waltham, MA, USA). Reverse transcription reactions were performed on 1000 ng of total RNA using the High Capacity cDNA Reverse Transcription Kit (Thermo Fisher Scientific) according to the manufacturer’s protocol. cDNA was stored at −20 °C.

### 2.4. Quantitative Real-Time PCR Analyses

RT-qPCR reactions were performed as described previously [33]. The SYBR Green-based system (Promega, Madison, WI, USA) was used by absolute quantification using the standard curve method. Specific primer pairs for the chondrogenic and DNA methylation-associated genes were designed by the Primer-BLAST service of NCBI and ordered from Integrated DNA Technologies (IDT, Coralville, IA, USA). Nucleotide sequences of the primer pairs are shown in Table S2 in the Supplementary Materials. Conventional PCR was used to create standard curves for absolute quantification. The Promega GoTaq Flexi DNA Polymerase kit (Promega, Madison, WI, USA) was applied to set up the following mixture (50 μL per each reaction): 1.25 U GoTaq polymerase; 3 mM MgCl_2_; 0.2 mM dNTP; 200 nM primers; and 50 ng cDNA (pooled from 6-day-old untreated samples). Amplification was performed in a programmable thermal cycler (MultiGene 96-well Gradient Thermal Cycler; Labnet International, Edison, NJ, USA) using the following thermal profile: initial denaturation (95 °C, 5 min), followed by 40 cycles of denaturation (95 °C, 15 s), annealing (58 °C, 20 s), extension (74 °C, 20 s), and final extension (74 °C, 5 min). A Roche High Pure PCR Product Purification Kit (Roche, Basel, Switzerland) was used to purify the PCR products according to the instructions of the manufacturer. The DNA concentration of the isolated PCR products was detected using a Nanodrop 1000 UV-Vis spectrophotometer (Thermo Fisher Scientific). Purified samples were diluted in a serial manner (10-fold, starting with 1 ng/μL) to establish the standard curves. The QuantStudio 3 Real-Time PCR System (Thermo Fisher Scientific) was applied for the reactions using the GoTaq qPCR Master Mix (Promega) and 10 ng cDNA for each 10-μL reaction. The settings for the process were the following: initial denaturation (95 °C, 2 min), followed by 40 cycles of denaturation (95 °C, 5 s), annealing and extension (60 °C, 30 s), and final extension (72 °C, 20 s). Amplification was followed by a melt curve stage consisting of 3 steps: denaturation at 95 °C for 15 s, annealing at 55 °C for 15 s, and a dissociation step at 0.15 °C/s increments between 55 and 95 °C. Amplification data were analyzed using the QuantStudio Design and Analysis Software (version 1.5.1) and exported data were processed using Microsoft Excel. As for the cell line-based micromass cultures, the 2^−ΔΔCt^ method was used for data analysis and the measured Ct values were normalized onto the most stably expressed reference gene and the respective control (i.e., day 0). As for primary chondrifying micromass cultures, after calculating absolute quantities by comparing fluorescence intensity values to those of the standard curve, the expression data of genes of interest were normalized onto the expression data of the most stably expressed reference gene (as determined by the NormFinder algorithm; https://moma.dk/normfinder-software, accessed on 8 August 2021) and then onto the respective control (i.e., vehicle control or day 0), which was set at 1.00.

### 2.5. Quantitative Methylation-Specific PCR Analyses

Genomic DNA purification and subsequent bisulfite conversion of the template was conducted by an EZ DNA methylation-direct^TM^ kit (Zymo Research) following the company’s manual. DNA methylation was assessed by quantitative methylation-specific PCR (qMSP). qMSP primers were designed using MethPrimer 2.0 software and tested in pilot DNA methylation profiling assays. TATA box binding protein (TBP) promoter served as a negative control for methylation profiling assays since it is never methylated. These TBP promoter-specific unmethylated MSP primers were employed for normalization of qPCR data sets. Positive control primers for DNA methylation were the 3′ terminal exonic region of the *Prickle1* gene. Control and chondrogenic marker-specific qMSP primer sequences are provided in Table S3. qMSP assays were performed in a CFX96 PCR machine (Bio-Rad) and qMSP data sets were processed by CFX manager software.

### 2.6. Digoxigenin-Labelled RNA Probe Preparation

PCR primers were designed to amplify a ~1000-bps-long region from the 3′UTR of the *Dnmt3a*, *Ogt*, and *Tet1* genes. PCR-amplified 3′UTR regions were cloned into pDrive vector (Qiagen, Germantown, MD, USA) and sequenced. Insert-flanking T7 promoters were used for generating antisense probes. Sequence data of the cloned regions are given in Table S4 in the Supplementary Materials. The specific gene products of the *Dnmt3a*, *Ogt*, and *Tet1* probes were amplified with the help of PCR from the plasmids. Amplifications were performed in a thermal cycler (Labnet MultiGene™ 96-well Gradient Thermal Cycler; Labnet International, Edison, NJ, USA) using the following settings: 95 °C, 2 min, followed by 33 cycles (denaturation, 95 °C, 15 s; annealing for 20 s at 57 °C; extension, 72 °C, 75 s), and then 72 °C, 2 min. Digoxigenin-labelled RNA probe preparation was performed as recommended by Roche, with some modifications. The amplified PCR products were isolated using a Roche High Pure PCR Product Purification Kit (Roche, Basel, Switzerland) according to the instructions of the manufacturer. DNA concentration of purified PCR products were detected with the help of a Nanodrop 1000 UV-Vis spectrophotometer (Thermo Fisher Scientific). The specific RNA labelling was developed with a DIG RNA labelling mix by in vitro transcription of DNA. First, the following components were mixed together to create the DIG RNA labelling mix: 1 µL of purified PCR product (concentration between 100 and 200 ng/µL); 2 µL of 10× concentrated DIG RNA Labelling Mix (Promega); 4 µL 5× Transcription Buffer (Promega); 2 µL 100 mM Dithiothreitol (DTT) (Promega); 2 µL T7 RNA Polymerase (Promega), and 9 µL nuclease-free water (NFW) (Promega) to make a total reaction volume of 20 µL. After the components were mixed together, and the mixture was incubated for 2 h at 37 °C. Polymerase reaction was terminated by 2 µL 0.2 M EDTA (pH 8.0). The labelled RNA was precipitated after the addition of 2.5 µL 4 M LiCl and 75 µL pre-chilled 100% ethanol. After a brief mix with a vortex, the precipitate was incubated at −80 °C overnight. On the next day, the sample was centrifuged at 13,000× *g* for 15 min at 4 °C. The supernatant was discarded, and the pellet was washed with 100 µL of ice-cold 70% (*v*/*v*) ethanol. The precipitate was centrifuged again at 13,000× *g* for 15 min at 4 °C, and after discarding the supernatant, the sample was left to dry at room temperature for some minutes. Finally, the RNA pellet was dissolved in 75 µL of hybridization buffer (containing 20× saline sodium citrate (SSC), dextran sulfate, 50× Denhardt’s solution, sodium dodecyl sulfate (SDS), tRNA, and 50% (*v*/*v*) formamide; Sigma-Aldrich) and stored at −20 °C.

### 2.7. In Situ Hybridization

Whole murine embryos were collected as previously described. Briefly, NMRI mice were mated overnight, and detectable vaginal plug confirmed on the following morning, which was regarded as day 0. On gestational day 15, whole mouse embryos were retrieved from the uterus, washed in DEPC-PBS (PBS with 0.1% dietyhl-pyrocarbonate), and fixed in 4% paraformaldehyde (PFA, dissolved in DEPC-PBS) overnight. On the following day, embryos were washed in DEPC-PBS two times for 10 min each, then immersed into 15% and 30% RNAse-free sucrose solution until they sank. After embedding the embryos into Cryomount medium (Bio-Optica, Milan, Italy), 20-µm-thick frozen sections were cut in a sagittal plane using a cryostat (CM3050 S, Leica Biosystems, Buffalo Grove, IL, USA) and mounted onto Superfrost glass slides (Thermo Fisher Scientific). Sections were stored at −20 °C. We applied a nonradioactive in situ hybridization protocol described earlier, with some modifications [34]. Briefly, sections were removed from −20 °C and left at room temperature for 20 min. The glass slides were placed into a 58 °C incubator overnight for drying. On the following day, slides were removed from the incubator and left at room temperature for 20 min. Samples were fixed in 4% PFA (dissolved in DEPC-PBS) for 20 min. After washing with DEPC-PBS for 2 × 10 min, the remaining liquid was blotted, and samples were treated with 100 µL of Proteinase K solution (20 µg/mL; Promega) at 37 °C for 20 min. The slides were washed with DEPC-PBS for 2 × 5 min. Samples were prehybridized for 4 h at 58 °C, then the solution was changed to the hybridization solution that contained the RNA probe (1‒2 µg/mL) and the slides were incubated at 58 °C for 16 h. All components were RNAse free until this step. On the third day, slides were washed in 1× SSC at 58°C for 15 min, then in 1.5× SSC for another 15 min at 58 °C, and finally twice in 2× SSC for 2 × 20 min at 37 °C. Samples were treated with 0.5 µg/mL RNAse A dissolved in 2× SSC at 37 °C for 20 min. After washing in 2× SSC at room temperature for 10 min, slides were washed twice in 0.2× SSC at 58 °C for 2 × 30 min. Then, sections were washed twice at 58 °C for 2 × 15 min, then at room temperature for 10 min with PBST. Finally, samples were incubated in 10% Blocking buffer solution (Blocking buffer powder dissolved in maleic acid buffer with Tween (MABT); Roche) with α-DIG antibody (anti-digoxigenin, 1:1000; Abcam, Cambridge, UK; Cat. No.: ab420) at 4 °C overnight. Sections were then washed three times in PBT (PBS with 0.1% Triton X-100 and 2 mg/mL BSA) for 3 × 20 min, then twice in 1 M TRIS solution (pH 9.0) for 2 × 5 min. Digoxigenin antibody was visualized by incubation with TRIS-NBT/BCIP solution (20 mg/mL stock solution of nitro blue tetrazolium and 5-bromo-4-chloro-3-indoyl phosphate, dissolved in 1 M TRIS; Sigma-Aldrich) at room temperature in the dark for 2~20 h (depending on the amount of RNA). After the incubation time, samples were washed in PBST for 2 × 10 min. Finally, slides were mounted with DPX medium (Sigma-Aldrich). Photomicrographs of the sections were taken using an Olympus BX53 camera on a Nikon Eclipse E800 microscope (Nikon Corporation, Tokyo, Japan). The photomicrograph of a negative control section (where no specific RNA probe was used) can be found in Figure S1 in the Supplementary Materials. The captured images were analyzed with Image J (NIH, ver. 1.46). Relative optical density values were calculated by the calibration of absolute mean grey data on each sample (representative results were obtained from 6 independent normalized measurements). The calculated relative optical density values can be found in Figure S2 in the Supplementary Materials.

### 2.8. Dimethyl-Methylene Blue Staining Method

The dimethyl-methylene blue (DMMB) staining method was used to demonstrate the amount of metachromatic cartilage ECM in whole mouse embryos and also in primary chondrifying micromass cultures. Sections of whole embryos stained with DMMB served as a control for in situ hybridization. Frozen sections were prepared as described above. After the glass slides were removed from −20 °C, they were dried at room temperature for 10 min, then at 58 °C for 1 h. After washing in distilled water for 2 × 10 min, samples were stained with 0.1% (*w*/*v*) DMMB (Sigma-Aldrich) dissolved in distilled water for 5 min. Surplus dye was removed by washing the sections with distilled water for 3 × 10 min. Slides were mounted with DPX. Photomicrographs of the stained samples were taken as described above. As for micromass cultures, 30-μL droplets of the cell suspensions were inoculated on the surface of 10-mm round coverglasses (Menzel-Gläser, Menzel GmbH, Braunschweig, Germany) into 24-well culture plates. On day 4 or 6 of culturing, colonies were rinsed with PBS and fixed in a 4:1 mixture of absolute ethanol and 40% formaldehyde. After rehydration in a descending series of ethanol, cultures were stained with 0.1% (*w*/*v*) DMMB dissolved in 3% (*v*/*v*) acetic acid (pH 1.8). Surplus dye was washed in acetic acid, then with distilled water. Finally, cultures were mounted with Aquatex (Sigma-Aldrich). Photomicrographs of the stained cultures were taken as described above. Photomicrographs were analyzed by using an internally developed MATLAB (Mathworks Inc., Natick, MA, USA) application. Cartilage nodules rich in metachromatic cartilage ECM were defined by an approximate range of values in the RGB color space and the pixels were counted.

### 2.9. Treatment with 5-azaCytidine

First, 5-azacytidine (5-azaC; Cat. No.: A2385; Sigma-Aldrich) was used to inhibit DNA methyltransferases and to consequently activate specific gene regions by triggering DNA demethylation [35,36]. Then, 5-azaC was dissolved in dimethyl sulfoxide (DMSO) at 10 mM and then applied at a final concentration of 10 µM for 72 h on culturing day 1 or 3. Primary chondrifying micromass cultures were harvested on the 4th or 6th day of culturing, according to the treatment protocol. Control colonies were treated with equal amounts of the vehicle (DMSO).

### 2.10. Mitochondrial Activity (MTT) Assay

Cell viability was monitored as previously described [29]. Briefly, 24-well plates were used for culturing of primary chondrifying micromass colonies. First, 25 µL of MTT reagent (3-[4,5-dimethylthiazolyl-2]-2,5-diphenyltetrazolium bromide; 5 mg/mL in PBS) were pipetted into each well on culturing day 4 or 6. Cells were incubated for 2 h at 37 °C. Following the addition of 500 μL of MTT solubilizing solution (10% Triton X-100 in 2-propanol), optical density was measured at 570 nm (Chameleon, Hidex Ltd., Turku, Finland). Measurements were carried out in 3 samples of each experimental group in 3 independent experiments. Optical density readings of the experimental groups were normalized to those of the vehicle controls and shown as percentage changes.

### 2.11. Cell Proliferation Assay with ^3^H-Thymidine Labelling

The rate of cell proliferation was examined as previously described [29]. Briefly, 1 μCi/mL ^3^H-thymidine (diluted from methyl-^3^H-thymidine; 185 GBq/mM, Amersham Biosciences, Budapest, Hungary) was added to the culture medium of primary chondrifying micromass cultures on day 3 or 5, 16 h before the end of treatments. After washing with PBS, proteins were precipitated with ice-cold 5% trichloroacetic acid, and washed with PBS again. Colonies were then air-dried for one week and radioactivity was counted by a liquid scintillation counter (Chameleon, Hidex). Measurements were carried out in 9 samples of each experimental group in 3 independent experiments. Scintillation counting data of the experimental groups were normalized to those of the respective controls and presented as percentage changes.

### 2.12. Statistical Analysis

All data are representative of at least three independent experiments. Data in figures is representative of the mean ± SEM (standard error of the mean) of a single experiment. With regard to RT-qPCR reactions, one representative data set is shown out of 3 parallel experiments showing similar trends, and data were normalized to beta actin (*Actb*, in case of the cell line-based micromass cultures) or Succinate Dehydrogenase Complex Flavoprotein Subunit A (*Sdha*, in case of primary chondrifying micromass cultures), as calculated by NormFinder. Statistical differences were determined using paired Student’s *t*-test or One-Way ANOVA with Tukey HSD and Mann-Whitney test. The specific differences were considered statistically significant if *p* < 0.05. Statistical significance is indicated by asterisks as follows: *p* < 0.05 = *; *p* < 0.01 = **; *p* < 0.001 = ***.

## 3. Results

### 3.1. Dnmt3a, Tet1 and Ogt Display Distinct Expression Patterns in Murine Chondrogenic Models

**Figure 1:**
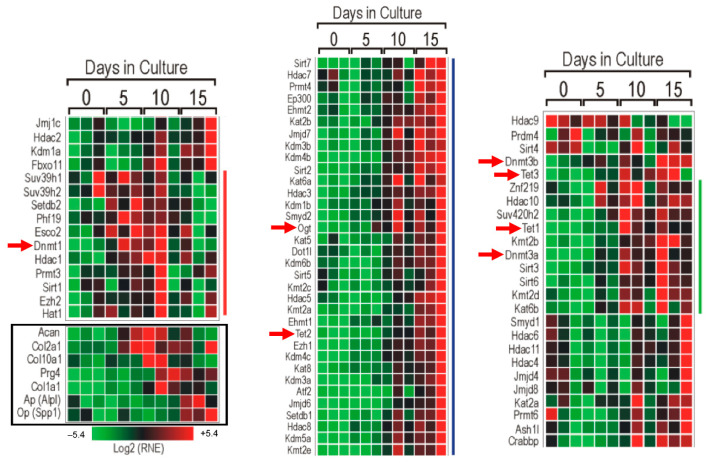
PCR array with micromass cultures established from C3H10T1/2 BMP-2 cells collected on designated days of in vitro cartilage formation. Chondrogenic differentiation-associated changes in the expression of epigenetic factors were visualized with a heatmap. The red squares refer to upregulated genes, and the green squares indicate downregulated genes. Genes next to the red line are mostly upregulated between the 5th and 10th days of culturing. Genes neighboring the blue line are upregulated around culturing day 15. Genes next to the green line are upregulated between the 10th and 15th days of culturing. Specific DNA methylation and demethylation regulator genes are marked with red arrows. Data indicated with the black rectangle: expressional changes of chondrogenic and osteogenic marker genes in order to verify the cartilaginous differentiation of micromass cultures.

We first studied the expression pattern of a set of epigenetic-associated genes in different in vitro murine chondrogenic model systems. Samples for PCR array were obtained from micromass cultures established from C3H10T1/2 BMP-2 cells collected on culturing days 0, 5, 10, and 15 (corresponding to the main stages of chondrogenesis in vitro), in order to examine the expressional peaks of epigenetic markers at the mRNA level. The results of the PCR array clearly showed the expression of every gene studied (Figure 1). Interestingly, many of the epigenetic-associated genes in connection with DNA methylation were upregulated at later stages of chondrogenic differentiation (culturing days 10 and 15). Three epigenetic modifiers were chosen for subsequent analysis: DNA methyltransferase 3 alpha (*Dnmt3a*), Tet methylcytosine dioxygenase 1 (*Tet1*), and O-linked *N*-acetylglucosamine (GlcNAc) transferase (*Ogt*), since the balance between *Dnmt3a* and *Tet1/Ogt* enzymes defines the actual methylation status of the genome (i.e., methylome). *Dnmt3a* was upregulated from culturing day 10, and it was strongly expressed on culturing day 15. *Tet1* expression peaked around day 10. It is worth noting that *Ogt*, which interacts with *Tet1*, displayed strong upregulation on culturing days 10 and 15. On the other hand, the expression profile of the chondrogenic markers collagen type II alpha 1 chain (*Col2a1*) and aggrecan (*Acan*) showed an earlier activation and increase in transcript levels between days 5 and 10 of culturing. *Col10a1*, a marker for matrix mineralization and chondrocyte hypertrophy, showed peak expression on days 10 and 15. The expression pattern of the *Col1a1* gene followed that of *Col10a1*, reflecting the initiation of osteogenic differentiation in the presence of hypertrophic chondrocytes.

**Figure 2:**
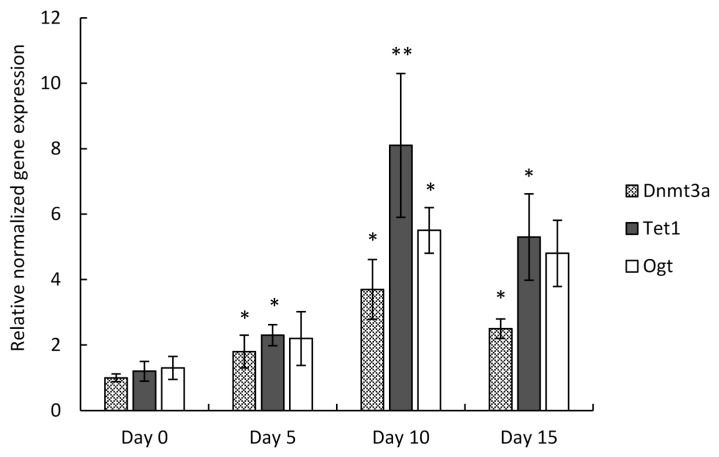
RT-qPCR analysis of *Dnmt3a*, *Tet1*, and *Ogt* gene expression in micromass cultures established from C3H10T1/2 BMP-2 cells, collected on culturing days 0, 5, 10, and 15. Measured C_T_ values were normalized to that of *Actb* and to culturing day 0. Mean ± SEM and levels of significance between consecutive culturing days (* *p* ˂ 0.05, ** *p* ˂ 0.01) are indicated. One-Way ANOVA with Tukey HSD was employed for evaluating significance. Representative results out of 3 independent experiments (biological replicates) showing similar trends of changes.

After RNA isolation from micromass cultures established from C3H10T1/2 BMP-2 cells, quantitative real-time PCR analysis was carried out to study the relative expression of the three genes involved in DNA methylation during chondrogenesis. The mean quantity values for the *Dnmt3a*, *Tet1*, and *Ogt* markers were normalized to *Actb*, and the fold-changes are relative to culturing day 0. All three genes displayed the largest increase of gene expression on culturing day 10 (*Dnmt3a*: 3.7-fold, ±0.91; *Tet1*: 8.1-fold, ±2.2; *Ogt*: 5.5-fold, ±0.7) (Figure 2). The relative gene expression of *Tet1* displayed the most prominent changes: the transcript level of *Tet1* indicated a significant elevation from culturing day 5 (2.3-fold, ±0.32), with the greatest degree of upregulation on day 10, and its mRNA level was still significantly high on culturing day 15 (5.3-fold, ±1.32). The expression profiles showed high similarity to those detected with the PCR array.

**Figure 3:**
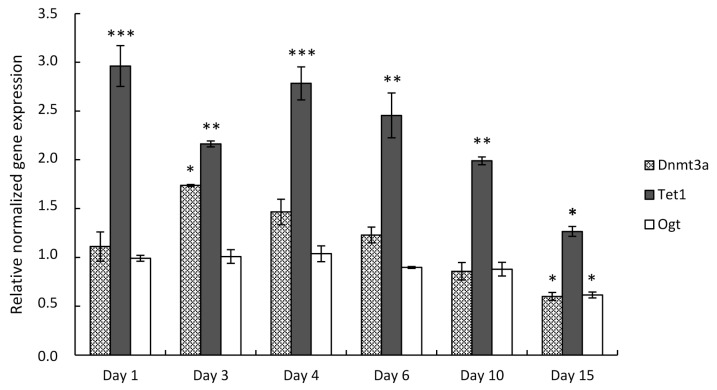
DNA methylation-specific marker gene expression in primary chondrifying micromass cultures on various days of culturing during in vitro cartilage formation as determined by RT-qPCR. Data are expressed as the mean ± SD relative to culturing day 0 and normalized against the reference gene *Sdha*. Statistically significant differences of gene expression levels between consecutive culturing days are indicated by asterisks as follows: * *p* < 0.05; ** *p* < 0.01; *** *p* < 0.001. Representative results out of 3 independent experiments showing similar trends of changes.

Next, we performed expression analysis of the genes of interest in primary chondrifying micromass cultures. Chondrogenic cell cultures were established from mouse embryonic limb buds and collected on designated culturing days. Transcripts for the DNA methylation genes were also identified in this in vitro model by RT-qPCR; however, their expression profile was more varied compared to the cell line-based model. After choosing the most stably expressed normalizing gene, the mean quantity values for the three examined DNA methylation-associated genes were normalized to the reference gene *Sdha*, and the normalized mean quantity was set to 1.0 on culturing day 0 for each of the genes. *Tet1* showed the highest expressional fold change among the three examined genes, with peaks on days 1 (2.96-fold, ±0.21) and 4 (2.78-fold, ±0.17) of culturing. The *Dnmt3a* transcript level was the highest on day 3 (1.74-fold, ±0.01) and displayed a significant downregulation by day 15 (0.6-fold, ±0.04). *Ogt*, on the contrary, was constantly expressed by the differentiating chondrocytes, except on day 15, when it was significantly downregulated (0.61-fold, ±0.03) (Figure 3).

**Figure 4:**
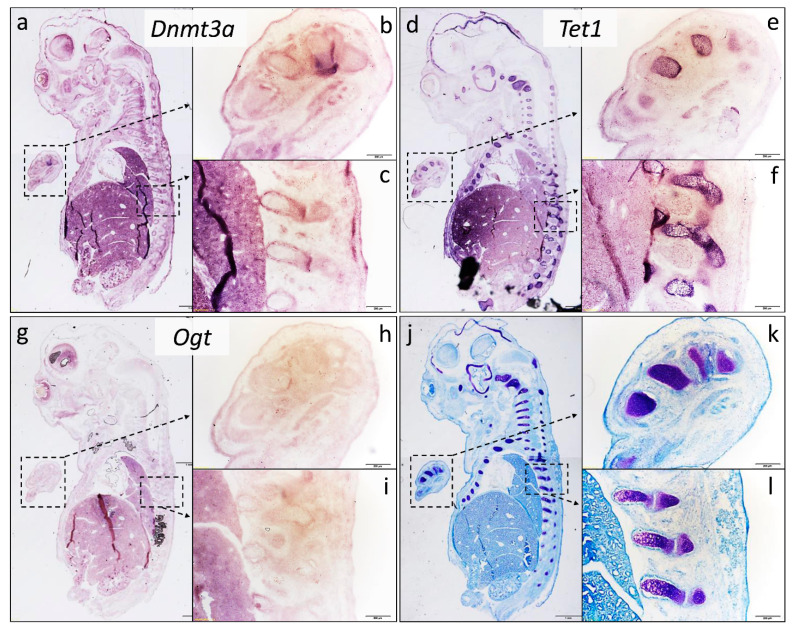
In situ hybridization analysis of epigenetic-associated gene expression in E15 whole mouse embryos. Sagittal sections of frozen embryos were processed with RNA probes encoding *Dnmt3a* (**a**–**c**), *Tet1* (**d**–**f**), and *Ogt* (**g**–**i**). Sections were also stained with DMMB for cartilage-specific proteoglycans (**j**–**l**). Metachromatic (purple) areas in photomicrographs show polyanionic glycosaminoglycan-rich cartilage ECM. Photomicrographs of sections from whole embryos were taken with a 4× objective (**a**,**d**,**g**,**j**). Inserts were taken with a 10× objective, which correspond to areas indicated with boxes (**b**,**c**,**e**,**f**,**h**–**k**). Note the strong expression of *Dnmt3a* and *Tet1* in maturing chondrocytes of the developing vertebrae and limb buds in the mouse embryo. Scale bar for (**a**,**d**,**g**,**j**): 1 mm, for the rest: 200 µm.

Furthermore, we aimed to prove the in vivo relevance of the three examined genes in chondrogenesis. Therefore, we performed in situ hybridization analyses of *Dnmt3a*, *Tet1*, and *Ogt* in 15-day-old whole mouse embryo samples. This experiment revealed that all three genes were expressed in the territories of hyaline cartilage islands of the developing forelimb, vertebrae, and sternum of the E15 mouse embryos. Among the three examined genes, densitometry analysis revealed that *Dnmt3a* showed a moderate expression pattern (Figure 4a–c), *Tet1* signals were the strongest (Figure 4d–f), while *Ogt* appeared to have a weak expression in the primitive limb and vertebrae compared to the other two epigenetic markers (Figure 4g–i; see also Figure S1 and Table S4 in the Supplementary Materials). At this developmental stage, cartilage had not started to differentiate into bone, the hypertrophic cartilaginous area was minimal, and ossification had not commenced yet, but the cartilaginous primordia of the future bones was well visible. The purple metachromatic color was obtained with the application of DMMB, in order to demonstrate the cartilage elements of the developing limbs, vertebrae, skull, and ribs (Figure 4j–l).

### 3.2. ECM Morphology, Cell Proliferation, and Cell Viability of Early and Late Chondrogenic Stages Are Different after 5-azaC Treatment

**Figure 5:**
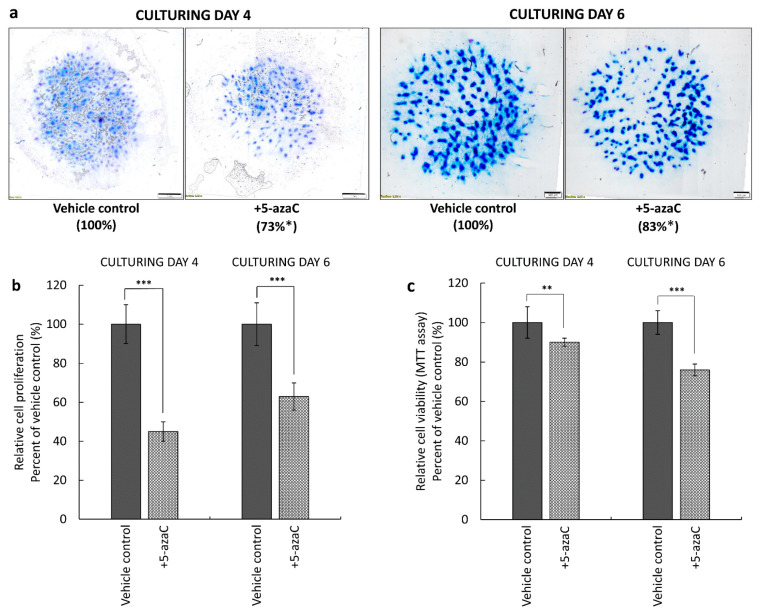
Effect of the DNA methylation inhibitor 5-azaC on cartilage ECM production, cell proliferation, and cell viability. (**a**) Metachromatic staining of 4- and 6-day-old primary chondrifying micromass cultures. 5-azaC (or DMSO as the vehicle control) was applied from the first or the third day of culturing, respectively, for 72 h at a final concentration of 10 µM. Metachromatic ECM accumulation was visualized by dimethyl-methylene blue (DMMB) qualitative staining assay, and the proportion of the metachromatic area was analyzed by MATLAB application (percentages are indicated under the photomicrographs). Original magnification was 4×. Scale bar: 1000 or 500 μm. Effects of 5-azaC on (**b**) cell proliferation and (**c**) cell viability (mitochondrial activity) in primary chondrifying micromass cultures. Cell viability was determined by using the MTT assay, and cell proliferation was examined by the ^3^H-thymidine incorporation assay on day 4 or day 6, following treatment with 5-azaC or DMSO (vehicle control). Statistically significant differences between the proliferation rate and mitochondrial activity of cells in cultures that received the inhibitor versus vehicle control cultures are marked by asterisks (* *p* < 0.05, ** *p* < 0.01, *** *p* < 0.001). Representative data out of 3 independent experiments.

In order to investigate the functional relevance of the three enzymes mediating DNA methylation, 5-azaC was applied on primary chondrifying micromass cultures at 10 μM. For each experiment, three micromass cultures (per biological replicate) were treated during the beginning of chondrogenesis (i.e., from day 1 for 72 h), while three cultures were treated from day 3 for 72 h to demonstrate its effects on later stages of chondrogenesis. To visualize cartilage-specific ECM accumulation in the primary chondrifying micromass cultures, the qualitative DMMB staining method was used on culturing days 4 and 6 at the end of the treatment protocols. The DNA methylation inhibitor significantly attenuated the amount of metachromatic ECM produced (to 73% of the control) when applied at the early stage of chondrogenesis. Interestingly, when 5-azaC was administered from culturing day 3 for 72 h, the morphology of metachromatic cartilage nodules was similar to that of the untreated micromass cultures. It is of note that in case of colonies treated at the late stage of differentiation, the characteristic metachromatic (purple) color was weaker (83% of the control) by day 6, indicating that the chondrocytes of these cultures probably produced somewhat less metachromatic ECM components (i.e., proteoglycans) compared to the controls (Figure 5a).

We hypothesized that one of the reasons behind the attenuated ECM production could be the altered proliferative and/or mitochondrial activity of the chondroprogenitor cells and chondrocytes. Thus, we examined the effects of 5-azaC on cell viability and cell proliferation during chondrogenic differentiation. The assays were carried out on culturing days 4 or 6, depending on the starting day of treatment. Both treatment regimens inhibited the proliferation of chondrifying cells, especially during the early stages of chondrogenesis, when this parameter was lowered by 55% (±5%), as opposed to later stages, when the rate of cell division was reduced by 37% (±7%) (Figure 5b). We also studied the potential cytotoxic effect of 5-azaC during in vitro cartilage formation. The percentage of viable cells in the 4-day-old colonies after treatment was 90% (±2%), compared to the control group, and this was a significant decrease. In contrast, cells in 6-day-old primary chondrifying micromass cultures showed a massive reduction in their mitochondrial activity (24 ± 3%) (Figure 5c).

### 3.3. Inhibition of DNA Methylation by 5-azaC Influences Chondrogenic Marker Gene Expression Depending on the Developmental Stage of Chondrogenesis

**Figure 6:**
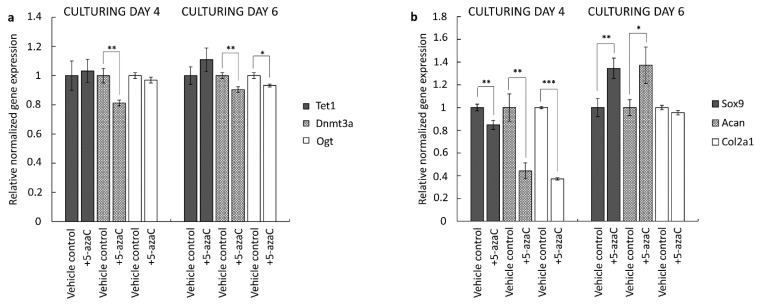
DNA methylation-associated (**a**) and cartilage-specific (**b**) gene expression in 4- and 6-day-old primary chondrifying micromass cultures after 5-azaC treatment (vehicle controls were treated with DMSO). The DNA methylation inhibitor was added to the culture medium from the first or the third day of culturing, respectively, for 72 h, at a final concentration of 10 µM. Data are expressed as the mean ± SD relative to the vehicle control and normalized against the reference gene *Sdha*. Statistically significant differences of the gene expression levels are indicated by asterisks as follows: * *p* < 0.05; ** *p* < 0.01; *** *p* < 0.001. Representative data out of 3 independent experiments.

In order to detect the effects of 5-azaC treatment on gene expression profiles in primary chondrifying micromass cultures, RT-qPCR reactions were performed. We collected samples for total RNA isolation on culturing days 4 or 6. Here, 5-azaC was applied for 72 h prior to the sample collection. First, we wanted to check whether the expression of the investigated genes mediating DNA methylation was altered after the application of the inhibitor. To this end, we assessed the quantitative expression profile of *Dnmt3a*, *Tet1*, and *Ogt*. Our results confirmed that 5-azaC treatment significantly downregulated the expression of *Dnmt3a* (0.81-fold with ±0.08 on day 4 and 0.9-fold with ±0.08 on day 6) and *Ogt* (0.93-fold with ±0.01 on day 6) compared to the control, while *Tet1* expression was not influenced. This pattern was similar in the two different experimental groups and reflected a transcriptional influence of 5-azaC on the *Dnmt3a* and *Ogt* genes (Figure 6a).

Next, we studied the mRNA levels of key chondrogenic marker genes with RT-qPCR. The key chondrogenic transcription factor *Sox9*, as well as the two major cartilage matrix-specific genes (*Col2a1* and *Acan*) were selected. We found that the expression profiles of these genes were significantly altered after the inhibition of DNA methylation at both the early and the late stages of chondrogenesis (Figure 6b). During the early stage of in vitro cartilage formation, all three marker genes were significantly downregulated. The largest decrease was detected for *Col2a1* (0.37-fold, ±0.01) and *Acan* (0.44-fold, ±0.07). On the contrary, during the later stage of chondrogenesis, *Sox9* (1.35-fold, ±0.09) and *Acan* (1.37-fold, ±0.16) were significantly upregulated, while *Col2a1* expression remained unchanged.

**Figure 7:**
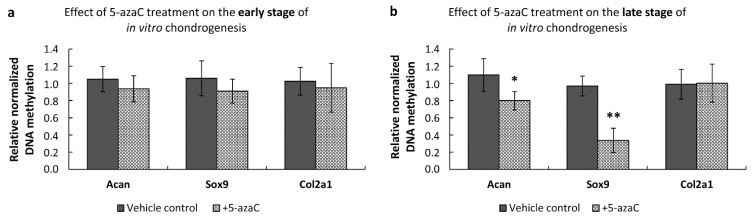
Methylation status of the promoters of cartilage-specific marker genes in primary chondrifying micromass cultures after 5-azaC treatment. (**a**) Changes of the DNA methylation profiles during the early stage of chondrogenesis, where 5-azaC was administered from the 1st day of culturing for 72 h, and cultures were harvested on culturing day 4. (**b**) Changes in DNA methylation during the late stage of chondrogenesis: 5-azaC was applied from the 3rd day of culturing for 72 h, samples were harvested on culturing day 6. TATA box binding protein (TBP) promoter served as a negative control, and the qPCR data sets were normalized against the TBP promoter-specific unmethylated MSP primers. Data are expressed as the mean ± SEM. Statistically significant differences of methylation levels are indicated by asterisks as follows: * *p* < 0.05; ** *p* < 0.01. One-Way ANOVA with Tukey HSD was employed for evaluating significance.

To investigate whether 5-azaC treatment had a direct effect on the observed gene expression changes of the *Col2a1* and *Sox9* genes, we conducted quantitative methylation-specific PCR on isolated genomic DNA samples. We found that the DNA methylation profiles of the investigated promoters (i.e., *Acan*, *Sox9*, and *Col2a1*) were not affected in the early chondrogenic phase (Figure 7a). However, 5-azaC-mediated inhibition during the late phase of chondrogenesis significantly decreased DNA methylation in *Acan* (0.8-fold, ±0.107) and *Sox9* (0.34-fold, ±0.141) promoters, which could explain the observed altered gene expression of these two genes (Figure 7b).

## 4. Discussion

Recent studies indicate that DNA methylation may serve as a promising therapeutic target for several human joint disorders, including osteoarthritis [37]. Since the stem cell therapy-based approach represents a very attractive component in the toolkit of regenerative medicine, a better understanding of DNA methylation during early chondrogenesis is essential. To this end, we investigated the temporal expression pattern of specific regulators of DNA methylation at the mRNA level in different murine chondrogenic models, and studied the effects of the DNA methylation inhibitor 5-azaC on chondrocyte differentiation.

First, we looked at the osteo-chondrogenic differentiation in micromass cultures established from C3H10T1/2 BMP-2 cells [38]. The cell line-based micromass cultures were collected for RNA isolation on designated days of culturing, based on the specific differentiation stage of chondrocytes in vitro: the phase of proliferation occurs between days 0 and 3 (with mostly chondroprogenitor cells and early chondroblasts present in the micromass culture), and also the phase of differentiation that takes place between days 3 and 6 (with chondroblasts and mature chondrocytes that produce a high amount of cartilage-specific ECM). After culturing day 6, mature chondrocytes transform into hypertrophic chondrocytes, and this process leads to an intense calcification of the micromass culture [39,40]. In terms of the chondrogenic marker expression patterns, the results of the PCR array showed good correlation with our earlier study, which analyzed the transcript levels of the same markers by conventional RT-PCR [31]. The proteins coded by the *Col2a1* and *Acan* genes are characteristic components of the cartilage-specific ECM [41]. According to the PCR array, these genes were upregulated around the fifth day of culturing, corroborating our earlier results suggesting that metachromatic areas corresponding to glycosaminoglycan-rich cartilage ECM started to appear from the third day of culturing [31].

After verifying the expression of chondrogenic marker genes by the PCR array in murine cell line-based micromass cultures, we undertook analysis of the gene expression profiles of several epigenetic markers using a PCR array. We selected three epigenetic-associated genes (*Dnmt3a*, *Tet1*, and *Ogt*) for further analysis as their balanced function is necessary for the actual methylation status of the genome. The importance of the *Dnmt3b* enzyme in normal limb development and hypertrophic chondrocyte maturation has already been proven [13]. *Dnmt3b* plays a significant role also in regulating cellular metabolic processes in postnatal articular cartilage [42]. This was visible with the PCR array, where the expression of the *Dnmt3a* and *Dnmt3b* genes showed strong elevation as chondrogenesis proceeded into later stages. TET enzymes contributing to the reversible nature of DNA methylation were also investigated, as recent studies pointed out that *Tet1* might be a key epigenetic regulator of chondrogenesis. Although lineage-specific knockdown of *Tet1* caused only minor skeletal abnormalities in transgenic animals, significant downregulation of the cartilage matrix-specific gene expression was observed by in vitro experiments [19,21,43]. In terms of the spatiotemporal distribution of TET enzymes in the developing vertebrae of mouse embryos, *Tet1* was the only protein that was detectable during chondrogenesis, from the appearance of chondroprogenitor cells until the hypertrophic transformation of mature chondrocytes between E14.5 and E16.5. Although *Tet2* was the most abundant protein, its expression level was the highest at E12.5, when cartilage formation was at the primordial stage, while *Tet3* expression was only positive at the beginning of osteogenesis at E18.5 [44]. In line with these observations, *Tet1*, *2*, and *3* showed intense expression in the PCR array during the second half of in vitro chondrogenesis.

In addition to the cell line-based model, we also employed a primary chondrifying micromass culture system established from murine limb bud-derived chondroprogenitor mesenchymal cells [45] to validate the expression profiles of the selected genes. In primary micromass cultures, moderately high *Dnmt3a* expression was detected at the time of the commitment of chondrogenic cells (i.e., day 3 of culturing), and a gradual decrease along with the progress of chondrogenesis was seen when the RT-qPCR results were analyzed. The expression of *Tet1* showed significantly elevated levels compared to the other two genes of interest. *Ogt*, encoding a molecular partner of TET enzymes, showed a low and constant level of expression as revealed with RT-qPCR. The reason behind the different quantitative gene expression profiles between the cell line-based and primary chondrifying micromass cultures could be attributed to the differences in the rate of differentiation, and the state of chondrogenic commitment of the cells in the cultures. The micromass cultures established from C3H10T1/2 BMP-2 cells demonstrated a distinct macroscopic morphology compared to the primary chondrifying micromass cultures on culturing day 6 according to our earlier results [31], suggesting that the chondrogenic differentiation of cell line-based micromass cultures might be slightly slower. One reason behind this phenomenon could be the different migratory ability of the differentiating cells: the embryonic limb bud-derived chondroprogenitor cells remained together during condensation and formed distinct prechondrogenic nodules in the primary chondrifying micromass cultures, while the peripheral cells of micromass cultures established from C3H10T1/2 BMP-2 were extremely migrant. Additionally, primary chondrifying micromass cultures represent a more heterogeneous cell population, in which a low number of fibroblasts can also be present. These data support the relevance of the simultaneous analysis of the genes of interest in cell line-based and primary chondrogenic models.

Next, we aimed to detect the expression of the three examined epigenetic-associated genes at the mRNA level in cryosections of 15-day-old whole mouse embryos by in situ hybridization. We chose the age of the embryo by considering the stages of cartilage development as described by Rafipay et al. [46]. They established that cartilage and bone formation in the developing limbs of a mouse embryo took place between E12.5 and E16.5. The first cartilaginous areas were identified in the forelimb at E12.5, while the mineralization-specific Alizarin red staining was recognizable from E15.5. Thus, we decided that E15 was the most suitable stage to examine the DNA methylation-related gene expression. At that stage, chondrocytes in mature hyaline cartilage could be easily identified, and the developmental stage of chondrocytes in E15 mouse embryos is almost the same as in the 6-day-old primary chondrifying micromass cultures. The in situ hybridization results were in good agreement with the findings of the quantitative real-time PCR studies. The most important similarity between the results of in situ hybridization and RT-qPCR was that in both cases, *Tet1* had the strongest expression pattern among the three examined epigenetic regulators. The significant role of this factor in osteochondrogenic differentiation was confirmed by experiments in which the specific knockout of *Tet1* impaired the skeletal development of the mutant mice: the examined animals showed growth irregularities or even embryonic lethality [47]. Specific knockdown of *Tet1* in the ATDC5 chondroprogenitor cell line also altered chondrogenic differentiation [12]. Similar effects were observed in C3H10T1/2 cells: downregulation of *Tet1* caused a decrease in chondrogenic markers, such as collagen type 2 [19].

We also investigated the effects of the DNA methylation inhibitor 5-azaC on chondrocyte differentiation. Based on data found in earlier studies [26,48,49], we applied the compound at a final concentration of 10 µM. This concentration was five-fold higher than that applied to mature chondrocytes by Duan et al. [50], but it was well tolerated by the chondrogenic cells as shown by the viability assay. The cell proliferation assay indicated that 5-azaC affected two important phases of in vitro chondrogenesis. We found that the early phase of chondrogenesis was more strongly affected by the treatment than the late phase of differentiation, causing an extreme loss in the proliferative ability of the cells during the first three days of culturing. An interesting conclusion of the RT-qPCR experiments was that 5-azaC treatment significantly lowered the expression of the *Dnmt3a* gene during in vitro chondrogenesis in primary chondrifying micromass cultures, while the expression of *Tet1* gene was not altered.

**Figure 8:**
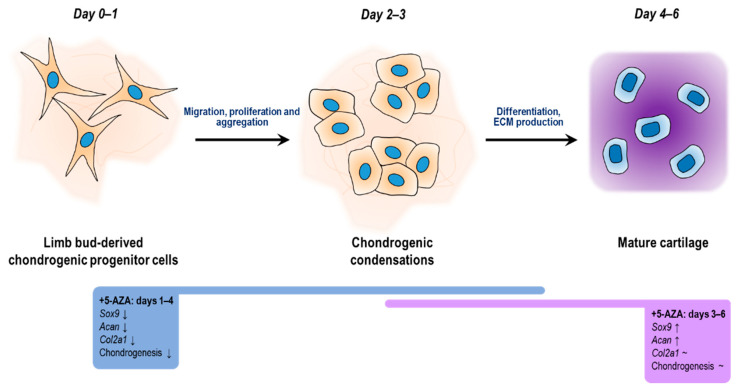
Diagrammatical representation of the key stages of chondrogenic differentiation of embryonic limb bud-derived micromass cultures, showing the regulation of chondrogenic genes triggered by the methylation inhibition 5-azaC on different culturing days.

Further, 5-azaC treatment has been documented to enhance the chondrogenic differentiation of human bone marrow-derived MSCs (hBM-MSCs) [51] and adipose-derived stem cells (ASCs) [52], and it also augmented the proliferation activity of ASCs [52]. While BM-MSCs retained their multipotent capacity after one pulse with 5-azaC, additional pulses resulted in a restricted differentiation potential with a concomitantly increased tendency for chondrogenic commitment [53]. However, in a different study, the expression of chondrogenic marker genes was reported to be negatively affected in chondrogenic cell cultures established from undifferentiated hBM-MSCs that were stimulated with 5-azaC for 24 and 48 h [17]. The controversies between our results and those reported by others may be explained by the specific differentiation state and origin of MSCs, and the duration and timing of 5-azaC delivery [54]. Here, we demonstrated that 5-azaC exerted a differentiation stage-dependent effect during in vitro hyaline cartilage formation in the primary chondrifying micromass model. The mRNA expression levels of *Sox9*, *Col2a1*, and *Acan* significantly decreased when 5-azaC was applied during the early stages of chondrogenesis; however, we could not detect significant hypermethylation in the promoter regions of the three chondrogenic marker genes examined, implying that the treatment altered the expression patterns indirectly. It can be hypothesized that 5-azaC treatment could have activated genes encoding repressor proteins involved in the downregulation of *Sox9*, *Col2a1*, and *Acan* genes. Nevertheless, 5-azaC-mediated blockage of DNA methylation at a later stage of chondrogenesis induced increased expression of the *Sox9* and *Acan* genes. The observed upregulated gene expression could be traced back to hypomethylation in the corresponding promoters, indicating that DNA methylation directly controls the transcriptional activity of key factors of chondrogenesis. Figure 8 summarizes the results presented in this study of how 5-azaC treatment influenced in vitro chondrogenesis.

Our study has some limitations. First, whilst we analyzed the expression profiles of genes encoding enzymes mediating DNA methylation/demethylation, and the methylation status of chondrogenic marker genes following 5-azaC treatment, performing a de-tailed genome-wide methylation analysis was beyond the scope of the present work. Future studies aimed at analyzing the methylome of chondrogenic cells are necessary. Second, although the employed murine chondrogenic models are widely accepted, the results obtained using rodent cells may not be directly applicable to humans. Nevertheless, since the results described above are similar between the two different murine chondrogenic models, it is plausible to assume that they are transferable to other models. Future studies will need to confirm the expression patterns of these genes during cartilage formation in humans.

## 5. Conclusions

This is the first study to report the differentiation stage-dependent transcript expression patterns of key enzymes known to mediate DNA methylation and demethylation during in vitro chondrogenesis of primary chondrifying micromass cultures. Both enzyme systems showed increased gene expression patterns during the early and the middle stages of chondrogenic differentiation, which was followed by a gradual decrease at the later phase. The two epigenetic mechanisms (i.e., DNA methylation and demethylation) are mutually exclusive; however, considering different regulatory regions (i.e., promoters and enhancers), the two processes can also take place simultaneously. The differentially methylated regions must be identified in order to provide better insight into the epigenetic regulation of chondrogenesis. The differentiational stage-dependent effects of 5-azaC on chondrogenic cells suggest the need of careful design for research application of this compound [54]. Moreover, 5-azaC can also inhibit RNA methylation, which might provide another regulatory layer for chondrogenic differentiation [55]. Therefore, it is reasonable to consider that option when the effect of 5-azaC treatment is evaluated.

## Data Availability

The data presented in this study are available on request from the corresponding author.

## Supplementary Materials

The following are available online at https://www.mdpi.com/article/10.3390/cells10102678/s1, Table S1: Sequences of primer pairs used for the PCR array analyses, Table S2: Sequences of primer pairs used for the RT-qPCR reactions, Table S3: Sequences of primer pairs used for the qMSP analyses, Table S4: Sequence data of the 3′UTR regions of *Dnmt3a*, *Ogt* and *Tet1* genes with insert flanking T7 promoters for antisense probe preparation, Figure S1: Photomicrograph of an E15 mouse embryo used for in situ hybridization as a negative control (no specific RNA probe was used), Figure S2: Quantitative (relative optical density) values of the *Dnmt3a*-, *Tet1*- and *Ogt*-specific in situ hybridization photomicrographs.
