# Supplementary material for: Analysis of Gene Expression Patterns of Epigenetic Enzymes Dnmt3a, Tet1 and Ogt in Murine Chondrogenic Models
Source: Cells. 2021 Oct 6;10(10):2678. doi: 10.3390/cells10102678 (PMC8534543; doi:10.3390/cells10102678)
Supplement: Supplementary file 1 [file cells-10-02678-s001.zip › cells-1393677-supplementary.pdf]

Supplementary Materials for

Analysis of gene expression patterns of epigenetic enzymes *Dnmt3a*, *Tet1* and *Ogt* in murine chondrogenic models

Judit Vágó, Katalin Kiss, Edina Karanyicz, Roland Takács, Csaba Matta, László Ducza, Tibor A. Rauch, Róza Zákány*

#Corresponding author. E-mail: roza@anat.med.unideb.hu

This PDF file includes:

- Sequences of primer pairs used for the PCR array analyses.
- Sequences of primer pairs used for the RT-qPCR reactions.
- Sequences of primer pairs used for the qMSP analyses.
- Sequence data of the 3'UTR regions of *Dnmt3a*, *Ogt* and *Tet1* genes with insert flanking T7 promoters for antisense probe preparation. These regions were cloned into pDrive vector and later amplified for digoxigenin-labelled RNA probe preparation.
- Photomicrograph of an E15 mouse embryo used for *in situ* hybridization as a negative control (no specific RNA probe was used).
- Quantitative (relative optical density) values of the *Dnmt3a*-, *Tet1*- and *Ogt*-specific *in situ* hybridization photomicrographs. The values were calculated specifically for the areas of limb buds and vertebrae.

Table S1. Sequences of primer pairs used for the PCR array analyses. The table continues on the next page.

Epig.Modif.	Gene	Forward	Reverse
DNA methylation	**Dnmt1**	CAGAGGAGAGAGACCAGGATAA	GCTGTTACCTCTTCCAGTTTCT
	**Dnmt3a**	CAAGGGACTTTATGAGGGTACTG	TTCTCAAAGAGCCAGAAGAAGG
	**Dnmt3b**	GAACCCTGGAGCTGCTATATG	CAACTTGGGTGGCTCAAATTC
	**Tet1**	CCAAGTGGGTGATCAGAAGAA	CACAGCAGGATAAGGACAACTA
	**Tet2**	AGGATGCAATCCAGACAAAGA	GCTTTACCCTTCTCTCCATACC
	**Tet3**	GAACTCATGGAGGATCGGTATG	CAGTGTGTGTCTTCGGATCA
	**Ogt**	GACTGGCGACTACACAGATTA	GGGAACGGGTGGTTACAATA
Histone acetylation	**Atf2**	ATGGCAGTGGATTGGTTAGG	GAGAAGCCGGAGTTTCTGTAG
	**Crebbp**	CAACAAACCATCCTGGGATCT	GGGTCTATGGGATTTGGGTTAC
	**Ep300**	GGCAATGCTGGCAGTTTATT	TTCCCATCTTAAGTGGTTGGG
	**Hat1**	GCCTGAAGATCTTGCTGTACTA	TCATCTGCCTCCACACAATC
	**Kat2a**	GCTCTTGGGAATGGTAGTAGATG	GCAAGAGCTTGAAGAGGTAGAA
	**Kat2b**	GAGGAGACCTCCAGCAGATAAT	TGAGAAACGTGAGCAGCAAG
	**Kat5**	TTTCCCTCAGAATGGGTCAG	CATCAGTGCCCAAGCAATTAG
	**Kat6a**	CCCATCTGTAGCTTCTGTCTTG	CACGATGGATGACCGCTATT
	**Kat6b**	GTGCCGATCCCATTCCAATA	ACAGGATGGGTGTCCACTA
	**Kat8**	TCTATGTTCACTATGTGGGCTTTA	GCCAGCTCACTCAGGTATTT
Histone deacetylation	**Hdac1**	GATGCAGAGATTCAATGTTGGTG	GATGTCCGTCTGCTGCTTATTA
	**Hdac2**	CCTCATAAAGCCACTGCTGAA	CCGACGTTAAATCTCTGCATCT
	**Hdac3**	TGATGACTGCCCAGTGTTTC	GGCCCAGTTGATGGCAATA
	**Hdac4**	CTTGAGGGCCGCTTTATCA	GGAAAGAAACACAACCAAGTCTATC
	**Hdac5**	AGGAGGAGCTGGAGAAACA	GATGGCACTCTCTTTGCTCTT
	**Hdac6**	CCCAATCTAGCGGAGGTAAAG	GTTCAGATCCAGCCCTTGAA
	**Hdac7**	CAGAAGCTGGCTGAAGTGAT	GCTCAAGAGTTCTGTAGGGAATAC
	**Hdac8**	CCTTCCACACTGATGCCTATC	GGCAGTCATAACCTAGTCCATATT
	**Hdac9**	GGAGCACATCAAGGAACTTCTA	GCCTCTCTACTTCCTGTTCTTG
	**Hdac10**	AGACCCAGACCCTGGATAAA	AAAGGTGTCCGGGTGAAAG
	**Hdac11**	GAGCTGAAGTGGTCCTTTGT	TCCTCTGCACAAGGAAGTTG
	**Sirt1**	GGATCCTTCAGTGTCATGGTT	CACCGAGGAACTACCTGATTAAA
	**Sirt2**	CCAACCATCTGCCACTACTT	CGTGTCTATGTTCTGCGTGTA
	**Sirt3**	GCCCAATGTCACTCACTACT	GATCCCAGATGCTCTCTCAAG
	**Sirt4**	CGCTGCTCAAGATCCCTAAG	GGGCAGCTCTCATTTCTGTAA
	**Sirt5**	GTGTCTAGTGGTGGGAACATC	GGGTGGTCTCCATGTTAAACT
	**Sirt6**	CCCAAGTGTAAGACGCAGTA	CAGTCCAGAATGGTGTCTCTC
	**Sirt7**	GTGGTGTCTCAGAACTGTGATG	CAGGAGGTGCAGACTTCAATATAC

Table S1. Sequences of primer pairs used for the PCR array analyses.

Histone methylation	**Ash1l**	GAGAGAGGAACTTCGTGCTAAA	TGTACAGCGATGCTGAGTG
	**Dot1l**	CATCACTACGGAGTGGAGAAAG	ACCTCGTTCCAGTGTGTATTC
	**Ehmt1**	GTCTGACCTGAGTTCTGAATCC	CTGCTCGGCTTCTTTCTACTT
	**Ehmt2**	ACAACGCACGCCACTAAT	CATCCTCTTCCTTGCTGTAGAC
	**Ezh1**	GGAGCAAAGGCTCTGTATGT	GGCTGGACACGAAGTTTCT
	**Ezh2**	CATCCCGTTAAAGACCCTGAA	ACAGTTTCGTCTTCCACCATAA
	**Fbxo11**	GATGGATTTGCTGCAGGTATTG	GCCTCCAAATCGGTTGTTAAAG
	**Kmt2a**	CCAGACCCTCCTGTTCTTACT	CGCTGAACTCCAACACAGATAC
	**Kmt2b**	GCTGGACTGGAAAGCAGAA	CTAGGTCGCAATCCTCCTTAAA
	**Kmt2c**	TGTTAAGGCCTCACACCTTG	GGAGTGGACAAACTGCTTACT
	**Kmt2d**	GAGACATGTGACAAAGGGTATCA	CATAGCCGGCATGTCTTACA
	**Kmt2e**	AGCCCAGGAGTTTGGATAAAG	AGGAACTTCATCACCACTTTCA
	**Prdm4**	GACACACCAATAGAGAGCAGAG	ATGGAAGGACACCAACAACA
	**Prmt3**	AGACTTAAGAATCCTACGGTTGAA	GGAGTAGACACAGGCTCATAAAG
	**Prmt4 (Carm1)**	GGCGATTTGCACAGGATAGA	GGAGCCAATGAAAGCAACATC
	**Prmt6**	GGTGGAACAAGATACGGACAT	CTCATGGTCTCCCACTTTGTAG
	**Setdb1**	ATCAGCTCAAGATGTCCAGAAG	CCTGACCCAAGGTTCCTTTAT
	**Setdb2**	TCTGCCACAAATGGAGACTATG	CACTCTGAGTCACAGGTGTATG
	**Smyd1**	GGAAGTGGTGAAGGAGATGATAC	GCTTCACAACCTCGTGATACA
	**Smyd2**	CGGCGATATTCCCTGATGTT	TCCTGTACAGCTCTGACTTCT
	**Suv39h1**	CTCTGCATCTTCCGCACTAAT	CTGAGGTAATAATCTCTCCCACATAC
	**Suv39h2**	GTGAATCATAGTTGTGACCCAAATC	CAGCTCTTCTCCAGCGTTTAT
	**Suv420h2**	CTGCTCTCAAGACCCACATTT	AAGCACGGGTAGACACAATC
Histone demethylation	**Jmjd1c**	GCCATGATGCCAACAAGATATG	GTACAAAGAACCCTGGCAAATG
	**Kdm6b**	GAGGAACCAGACAGCACTAC	CTTCCACCTCTTGGCATCA
	**Jmjd4**	AAGTATGGAGACGCGGTTG	GTAACTGATATAGTCGCGGAAGG
	**Jmjd6**	CCATTGCCATCACCCAGAA	GCTCCTGTTTCAAGATCCTATACC
	**Jmjd7**	CTTGCACAAGGACCACTATGA	TGCTGGTGTGTAGAGATTGTAAG
	**Jmjd8**	GAATTGCAGGAGCTGGATCT	TGTCTTCTCAGGAGGGTAGAG
	**Kdm1a**	TGGGATCAGGATGATGACTTTG	GCACTGCTGTGTTCAGTTTAAT
	**Kdm1b**	GACTCAAATCTCCAGTGCAAAG	GATGGCACCTCTCTGTAGTATG
	**Kdm3a**	TCATCCACAGCAAAGACAGAA	GGCCTCTTGGATGAATCAGAA
	**Kdm3b**	GTGACCAAGCAGAGGATTCA	TTGCTCTTCTCCAACCTTCC
	**Kdm4b**	CAGCCGGATTACACAGGAAG	TGGACTCAGCGCAGTTAAAG
	**Kdm4c**	AGTTGTTATGGTGTCCCTTCTC	GAGCCCACTGATTGTTCTTAGT
	**Kdm5a**	CCTGAATGAACTTGAGGCAATG	CACCACAGGGATCTTCAATGTA
Chondrogenesis	**Acan**	TGGAGGTCATAGTGAAAGGTATTG	ATGATGGCGCTGTTCTGTAG
	**Col2a1**	TGGCTTCCACTTCAGCTATG	GGTAGGCGATGCTGTTCTT
	**Prg4**	TGGAGGACTAACAGGGAAGATA	CTGAATGTTGCCACCTCTCT
	**Alpl**	GCCATGACATCCCAGAAAGA	GCCAGACCAAAGATGGAGTT
	**Col1a1**	ACAAGGTGACAGAGGCATAAA	ACCAGGAGAACCAGGAGAA
	**Col10a1**	AACGCCCACAGGCATAAA	CTCCTCTTACTGGAATCCCTTTAC
	**Spp1**	CTTTCACTCCAATCGTCCCTAC	TGGCATCAGGATACTGTTCATC
	**Znf219**	CGGCCACCCAAGAAGAAA	CGCCGAGCGGAAAGATT

Table S2. Sequences of primer pairs used for the quantitative real-time PCR analyses. Nucleotide sequences, gene accession numbers and amplicon sizes are displayed for each primer pair.

Gene	Accession number	Primer sequence	Amplicon size
1. Epigenetic marker genes			
Dnmt3a	XM_006514953.3	CTGTCCCTACGACCAGTGC<br>CTGATGTCAAGCCCTCGGAA	110
Tet1	NM_001253857.2	CTGGGGCCATCCAAGTCAAT<br>TGTGTGAACCTGATTTATTGTGGT	120
Ogt	NM_139144.4	TTCAGTATTCTGTGCCGCCC<br>TCGTTGGTTCTGTACTGTCGG	188
2. Chondrogenic marker genes			
Sox9	NM_011448.4	GGAAGTCGGTGAAGAACGGA<br>AGATTGCCCAGAGTGCTCG	158
Col2a1	NM_031163.3	TCATCTTGCCGCATCTGTGT<br>TGCCCCTTTGGCCCTAATTT	164
Acan	XM_006540566.1	ATTCCCGCCCACCTACCT<br>GCTGACTAGGTTTCGGAGCA	190
3. Reference genes			
Actb	NM_007393.5	AGATCAAGATCATTGCTCCTCCT<br>ACGCAGCTCAGTAACAGTCC	174
Sdha	NM_023281.1	TCGACAGGGGAATGGTTTGG<br>GGACTCCTTCCGAGCTTCTG	110

Table S3. Sequences of primer pairs used for the quantitative methylation specific PCR (qMSP) analyses.

**Target name**		**Primer sequence**
M-spec.	TBP_F	GGGGATTCGTTGTAGAAGTC
	TBP_R	CGACATCAAATATACGTCAAACG
UM-spec.	TBP_F	GGGGATTTGTTGTAGAAGTTG
	TBP_R	AATAACAACATCAAATATACATCAAACATT
M-spec.	PRK_F	AGAAGTAGAAAGTTTCGGTTCGATA
	PRK_R	ACATAAACATACTAACTATAAAAATTAACG
UM-spec.	PRK_F	GGAGAAGTAGAAAGTTTTGGTTT
	PRK_R	CATAAACATACTAACTATAAAAATTAACA
M-spec.	Col2a_F	GGGATTGTAGATAATTTCGGG
	Col2a_R	ACAATCCCTAACCAACGATT
UM-spec.	Col2a_F	GGATTGTAGATAATTTTGGGG
	Col2a_R	CCAACAATCCCTAACCAACAAT
M-spec.	Acan_F	GTTTTAGGAAGAAGAATTTCG
	Acan_R	TACTCTATAATAAAACTAATAAATACCCGC
UM-spec.	Acan_F	GTTTTAGGAAGAAGAATTTTG
	Acan_R	CTCTATAATAAAACTAATAAATACCCACTC
M-spec.	Sox9_F	TAGATTTGTATATAGGTGGGCG
	Sox9_R	AAATTTAAATAAACACGCAACTTCG
UM-spec.	Sox9_F	AGATTTGTATATAGGTGGGTG
	Sox9_R	ATTTAAATAAACACACAACTTCAACT

Table S4. Sequence data of the 3'UTR regions of *Dnmt3a*, *Ogt* and *Tet1* genes with insert flanking T7 promoters for antisense probe preparation. These regions were cloned into pDrive vector and later amplified for digoxigenin-labelled RNA probe preparation.

>T7promoter-Dnmt3a (anti-sense) Clone#:D7
chr12:3,907,819-3,908,735

```
GCCAAGCTCTAATACGACTCACTATAGGGAAAGCTCGGTACCACGCATGCAGACGCGTTACGTATCGGATCCAGAATTCAGATTGCACG
CGAGTCTGGATAAATTCCACACTCAGCTTCCCCAGGGTCCGACCACCTCAGCTGAGCCCCTTTTTGGCAGGTGAAGCTAGGCATGGGGC
AGGGGTGGGGGGTGATATGGGTCATCCTTCAGTTTGTTAAAACCCCCTCCAGCTGCTCCCCTCTCCCCATCCTGGGCTGAAACCCTTTG
CACAGAGGGCCAGGCGGAAGCTGATGTCTTTGCTCATCTCGCTGTTTTGAAAGCACCATTATGAAGTCGGGATGTACAGTAGTTAACAG
TACCTTTTATATATATATATCTCATATCTATCATATATATATAAACTGTAAACAAGAGGTAACAGCGGCTTCTAGAAACTGGTTTTCTT
CAAGGTTTCCTGTGTGGTAGGCACCTGAAATACTGTAGAAAAGTGTCTGGTGTGGTGTTCTCTCAGCGCCCTGCTGCTAGTTGGGTTCT
GTTTTGCATGACCAGGAATGGTGCTGTTGAGACTGCTCGGGGGTGGGAGAGGAGACTCTGCCTCCGAGGGTCTCTATTCTCTTCCTGTT
TGTGCTCCTATCTGATCAGGCTAGAGACAACCAAAGAGATATATAGAAAAGGAAAAAAAAAAAACCAATATTTTAAGTTTTTATAGAAT
TTTCCCTCTCTCCCTTTCCTCTCCAAGGCATTTCTACTTTTTGTTGTTACTGATTTTGCTGCAATAACTCTCTTGTTTCAGTCACAGCA
AGAAACAAAAACCCAAACAAACAGAAAACCCCTCTGAAAAAGAGTAGAAAACAAAAAGTTCGCCTCCTAGGACCGGAGGGGAAGAAGGG
GAGGGGCCTATTTTGCTTTTTAGATAGGACTGAAGGTTAACATTGAAAACTCAGGAGATGATGTCCAACCCTTTTGCAAGGCCAAGCCC
TCCGGCGTTTTCCTCCTCTGGGGTTTGCTTTAAATTCCTTTTTCTCTTCTGGGTGCTGAACTTTTCTCCGTCCTCTCGTTCTTGGGGGT
TTAATGGTTTTGTTTTGTTTTTGGTTTGTTTGTTGGTTAAATTTTTTTATCATCACTACTTCAGTTTGCCCCCATGTCCCTTAACCACA
AATCACGAATTCGTCGAAAAGCTTCTCAAGCCTAGGCTAGCTCTAAACCCAACGTGTGGGGGCCCCAACTCCCGGCCCCTTGAATTCTA
TAAGGTCACCTAAAGGGCCGCACAATTCCATGGGCCGCCGTTTTACAACGCCGGGACTGGAAAAACCCTGGGGTTACCCAATTT
```

>T7promoter-Ogt (anti-sense) Clone#:O2
chrX:101,682,485-101,683,489

```
CCAAGCTCTAATACGACTCACTATAGGGAAAGCTCGGTACCACGCATGCAGACGCGTTACGTATCGGATCCAGAATTCGTGATTCTACC
AAGGCCGAATCCATAGAGTTTGAAGCTAAGTCCAGACATTCTAACACAGTCTAAAAATAAAATAACTGCCTGTATAGAGATGCTTCTTC
AGAGCGGTGAACACCATTTAAGGTACTGGAGAGAACTGTAATTTACTAACCAAACATTTGAATACTTATATACTTACCTGGGATTTCAT
TTCTGCTGAAAGAAATGGGAAGAACAGGACTCACTTTAAAAAAAAAAACAAACTTTAGAAAGGAAGGTAAAAATCTTATCACACTATTA
CTTTTGGAAGCAGCATAGGAGGGGCAGTCAAGGGCTGAAACACTGGTGAAACAGGTAAAACTTAGGCAGAAAAAAACCCTATATTATTA
TCAATAACAGTACCACAACTGTGACCTTCATGATTAATATTCACTCCTAAGAATTTTCATTTGGCACCAGATGGCATGTTCGAGAGAAA
CACCCTGGGATGTCTGAAAGCAGACATGGGTTTGTTAGCAGTCACAGGAGAATTTAACGGGAGCTGGGGTGTGTAAAACAGGCTGAAAA
GTCTTAAGTCATCATCTAAACAAAGCACTGACAGTGCCAAGCATTAGGTCAGACACTAAAATGACTGATATAGGCTCATGTGGTTTATA
AAACCTATAAAAAGACTACACCAGCAAGGTCCCTGTCACCCTGTCAGAGTTCAGACACTAAAACAGGAAGCAACATTTTAGTTTAAAAC
CTCAGAAGAATCAAGATCACTTCACATGAGTAAAAATACCTGAAAGCAAACATTTTTAAAAGCTCCAAAACCCAAAATATAAAGAAAAA
AAAAAGGATCAGAAGACTTGATCAATCCACGGCATCACAAAACGGCTGGTTAATCTCTATCTCCCCTCCACATTAAGCATCCCATGGAA
GACCAAGGGAGACCAGACCTTCCAGACCTGTGCACCACCTGTTTGCTGCAAAACTCTAAGAGATTCCTTGTGGAATAGCATTTCTTCTT
TCTTGTCTCTCCCTATCATGCAGAATCTGAATTCGTCGACAAGCTTCTCGAGCCTAGGCTAGCTCTAGACCACACGTGTGGGGGGCCCC
AACTCCCCGGCCGCTGTATTCCATAGGGGCCACCTAAATGGGCCGCACAATTCACTGG
```

>T7 prom-Tet1 (anti-sense), Clone#T1
chr10:62,811,427-62,812,547

```
CCAAGCTCTAATACGACTCACTATAGGGAAAGCTCGGTACCACGCATGCAGACGCGTTACGTATCGGATCCAGAATTCGTGATTGGGTA
AGGGTGACAATGCTAACGCAAGCATGTCCCCTCAATTATCTCTATAGAATATTTCCTTCTCATTTGGGAAGGACATGTCTCTTAATTAT
GTATTTTCTAATCTTGAAACACTGGGAACTTTTGACTGATCCCAATTTGCCTCCTTTTCCTTTGAAAACACCTGTAATTGGGTCATATA
GTTAATTGACGAGGTGACTTCAGAAGACAATATTTTTTCTAGTTAAAGAGGGAAAATTGATATTGGTAGCATCTTGTATTTAAATTTTT
TTTTTTAGAGACCCTCTATTCTATAAGTAAAGAACAGAAGCCGTGTGTTATTTGAGTACAGGCTGAGGTTAACGGGATGGGACTCTACA
GAGCAAGAACAAATGACTTCTCTGGTATTCAAATATGCACACATAATAGACAATAGGATTCTGCTACTATACTATTTCAAGTTACTCTT
AAAAGTAGAAAGTGCTTTTTAAAATATATACATTTATAGTTAGCGAACAGCTTCCAACCATCATGAGAATAGAGAACTACTTTATATAG
GAGAAATGTTTTAAGAGAAAAAGCATTTCATTGGTTTACTTCAAAGAAATGTGCAGACACGATGGCAATGATTGGAAGAGGCTTGAGCA
ATGGCACCAGAAAACAATCACTCAGCTCATCACTCCGTGTGTTGATGTGGTTAAAGCACCAGGAACTGTGTAGAGTTAACACTACATCT
TGGTCTTTACTTCTTATTGCAGGAACCTGTTATAAACCCTCTGAATAGTAAACAAACCCGGGGGAAAGCACCAGACTCTATGGTTCTAG
CATGGCCAACACCTAACCAAGATGTTCCTTTAAGTATTTAAAACTATAAAAAGTTTATTTTTGCTGAAAATGTTCACCTTCTACCAATT
CTCAAAATGTAATCACAGTTAAGTATTCACCCTTTTTTTTTTTTTTTTTAATATTTACGTCGGAATTGAAATGGGCGAAACAAAGCTTCC
GTAAACAACAAAATGACTTTCTTTTTAACAGCACCGGAAAAAACAAAAAAACCAAAGAAAACCAACCAACCAAACAAAAATTCCCCCTT
ACCCCCCTTTAGCCAAGGGCTTTACTTGGAGAAAAGCCTTTAGACCCACCGATTGGAAGGGAATCTGAATTCGTCCAAAAACTTTCCCA
AGCCTAGGCTAACTCTAAAACCAACCGTGGGGGGGGCCCCAACTTCCGGGCCCCTTGAAATCCTTAAGGGGCACCCTAAAGGGCCGCCC
AAATTTCCCGGGGCCCGCCTTTTTACAAGGCCCGGGACTGGGAAAAACCTGGGGGGTTACCAAATTAAAACCCCTTGGCGAAAAATCCC
CCTTTTCCCCCGTGGGGCGTATAAAGACAAGAGG
```

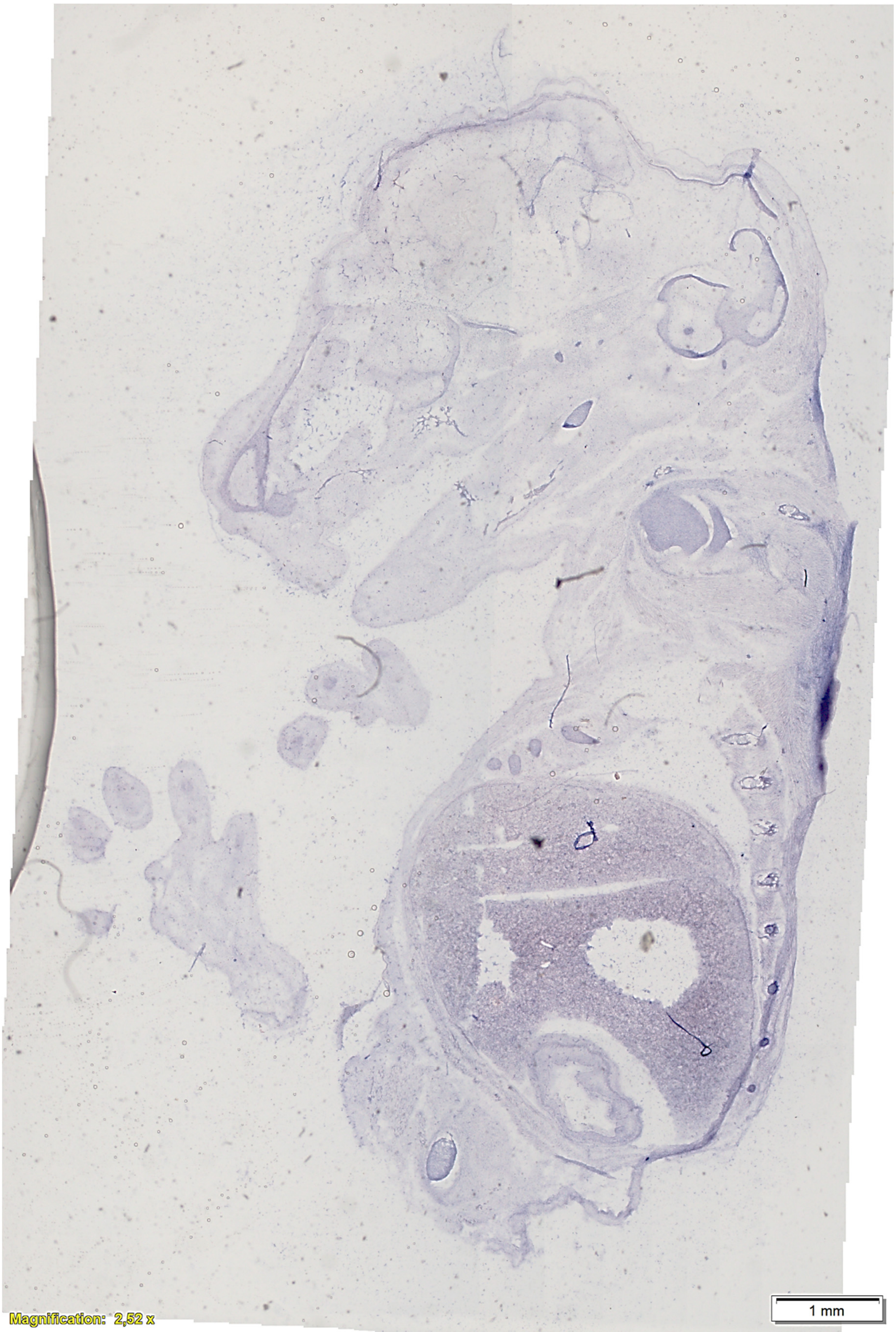

Figure S1. Photomicrograph of an E15 mouse embryo used for *in situ* hybridization as a negative control (no specific RNA probe was used).

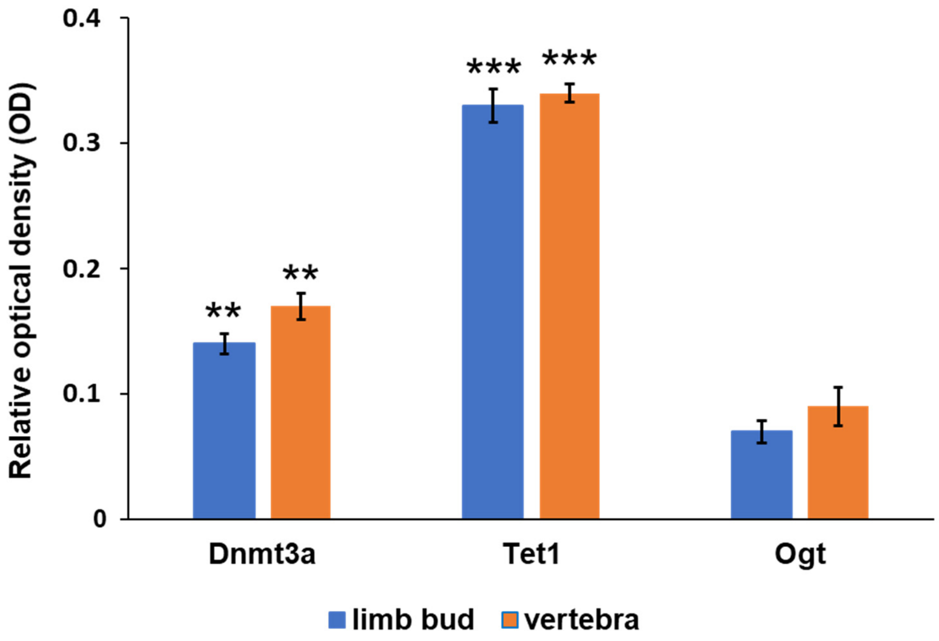

Figure S2. Quantitative (relative optical density) values of the *Dnmt3a*-, *Tet1*- and *Ogt*-specific *in situ* hybridization photomicrographs. The values were calculated specifically for the areas of limb buds and vertebrae. Mann-Whitney test was employed for evaluating significance. Statistical significance is indicated by asterisks as follows: $p < 0.01$ = **; $p < 0.001$ = ***.
